# Optimization of a 40-mer Antimyelin DNA Aptamer Identifies a 20-mer with Enhanced Properties for Potential Multiple Sclerosis Therapy

**DOI:** 10.1089/nat.2018.0776

**Published:** 2019-05-30

**Authors:** Brandon Wilbanks, John Smestad, Robin M. Heider, Arthur E. Warrington, Moses Rodriguez, L. James Maher

**Affiliations:** ^1^ Department of Biochemistry and Molecular Biology, Mayo Clinic College of Medicine and Science, Rochester, Minnesota.; ^2^ Medical Scientist Training Program, Mayo Clinic College of Medicine and Science, Rochester, Minnesota.; ^3^ Department of Neurology, Mayo Clinic College of Medicine and Science, Rochester, Minnesota.; ^4^ Department of Immunology, Mayo Clinic College of Medicine and Science, Rochester, Minnesota.

**Keywords:** aptamer, conjugate, multiple sclerosis, myelin, G-quadruplex

## Abstract

We previously reported the *in vitro* selection and characterization of a DNA aptamer capable of stimulating remyelination in a mouse model of multiple sclerosis. This aptamer was selected for its ability to bind to suspensions of crude murine myelin *in vitro*. Our initial studies *in vitro* and *in vivo* involved a 40-nucleotide derivative (LJM-3064) of the original 100-nucleotide aptamer. LJM-3064 retained robust myelin-binding properties. Structural characterization of LJM-3064 revealed that the guanosine-rich 5′ half of the sequence forms different G-quadruplex-type structures that are variably stable in the presence of physiologically relevant ions. We hypothesized that this structured domain is sufficient for myelin binding. In this study, we confirm that a 20-nucleotide DNA, corresponding to the 5′ half of LJM-3064, retains myelin-binding properties. We then optimize this minimal myelin-binding aptamer via systematic evolution of ligands by exponential enrichment after sparse rerandomization. We report a sequence variant (LJM-5708) of the 20-nucleotide myelin-binding aptamer with enhanced myelin-binding properties and the ability to bind cultured human oligodendroglioma cells *in vitro*, providing the first evidence of cross-species reactivity of this myelin-binding aptamer. As our formulation of DNA aptamers for *in vivo* remyelination therapy involves conjugation to streptavidin, we verified that the myelin-binding properties of LJM-5708 were retained in conjugates to avidin, streptavidin, and neutravidin. DNA aptamer LJM-5708 is a lead for further preclinical development of remyelinating aptamer technologies.

## Introduction

Multiple sclerosis (MS) is a central nervous system (CNS) demyelinating disease with increasing prevalence in the Western world, affecting ∼0.1% of the global population [1]. The etiology of MS is debated. The disorder is clinically characterized into four main subtypes: relapsing–remitting, primary progressive, secondary progressive, and clinically isolated syndrome [2]. Most MS patients are diagnosed with the relapsing–remitting subtype, and symptoms are often managed with anti-inflammatory agents, including interferon beta-1a [3], interferon beta-1b [4], glatiramer acetate [5], natalizumab [6], dimethyl fumarate [7], teriflunomide [8], and fingolimod [9].

Disease-altering therapy for progressive forms of MS has been less effective, however, with only modest clinical benefit observed for ocrelizumab (anti-CD20) [10], azathioprine (immunosuppressant) [11], glucocorticoids (immunosuppressant) [12], cyclosporine (immunosuppressant) [13], and interferon beta-1b (immunosuppressant) [14]. Although only 10% of patients have a diagnosis of primary progressive MS, by 25 years after diagnosis, ∼90% of patients with relapsing–remitting MS convert to the secondary progressive subtype. This makes the development of effective disease-altering therapies for progressive MS an important clinical priority.

The relative failure of immunosuppressive approaches to treat progressive MS suggests the need for other approaches to mitigate disease progression. Although many alternative therapeutic approaches have been proposed, regenerative therapies have emerged as one of the most promising avenues for future research. Example approaches include stem cell transplant to generate new oligodendrocytes [15], regulation of microglial activity in support of oligodendrocytes [16], and antibodies for stimulation of remyelination [17]. Each of these approaches seeks to take advantage of intrinsic regenerative mechanisms to achieve neuroprotection and regeneration for prevention of disease progression.

Inspired by reports of remyelination induced by natural human antimyelin immunoglobulin M (IgM) antibodies, we previously reported the application of systematic evolution of ligands by exponential enrichment (SELEX) using crude murine myelin as a selection target to yield a myelin-binding DNA aptamer (LJM-3064) capable of promoting remyelination in the Theiler's Murine Encephalomyelitis Virus model of MS [18]. In addition to its described remyelinating activity, LJM-3064 has a well-characterized G-quadruplex motif that exists in a structural equilibrium controlled by physiologically relevant monovalent ion concentration [19]. Further work has also shown that multivalent streptavidin conjugates of biotinylated LJM-3064 are rapidly distributed to the CNS and other tissues in mice [20].

When compared to IgM antibody-based approaches to stimulating CNS remyelination, DNA aptamer formulations are ∼10-fold smaller, more stable, and easier to synthesize thus offering many practical advantages [21]. Aptamer-based therapies have further significant advantages over treatment with antibodies and similar drugs, including the relatively low cost of oligonucleotide synthesis, high target specificity, and low potential for immunogenicity. In addition, aptamers are stable molecules that facilitate handling and long-term storage.

In the present work, we apply a rational approach to truncate the 40-nucleotide aptamer LJM-3064 to a 20-nucleotide minimal myelin-binding sequence, and perform an unbiased optimization of this minimal sequence using optimization SELEX. We report the emergence of an optimized sequence (LJM-5708) with enhanced myelin-binding properties relative to the parental aptamer, while also conserving the ability to bind to human oligodendroglioma (HOG) cells in culture. We further show that LJM-5708 retains the G-quadruplex properties of the parental aptamer, with formation of a parallel-stranded G-quadruplex structure stabilized by potassium cations. In addition, we show conserved myelin-binding properties of streptavidin, neutravidin, and avidin conjugates of LJM-5708 and assess the role each protein core may play in myelin binding. These studies lay the framework for future testing of optimized remyelinating aptamer formulations *in vivo*.

## Materials and Methods

### Optimization SELEX

A degenerate SELEX library based upon the 20-nucleotide minimal antimyelin aptamer LJM-5705 (G_3_TCG_2_CG_3_TG_4_TG_3_) was synthesized by Integrated DNA Technologies with 15% nucleotide randomization at each position, flanked by 5′ (AGAC_2_AGAC_2_AGCTGATAC_2_AGTCGTG) and 3′ (TACGC_2_A_2_GC_2_AC_2_TGCTC_2_TC_2_TGA) regions used for PCR amplification using forward primer 5′-FAM-AGAC_2_AGAC_2_AGCTGATAC_2_AGTCGTG-3′ and reverse primer (5′-A_20_-spacer18-spacer18-TCAG_2_AG_2_AGCAG_2_TG_2_CT_2_G_2_CGTA-3′). Optimization SELEX was then performed according to a previously described protocol with slight modification [18]. The degenerate library (300 nmol) was heated in phosphate-buffered saline (PBS) for 1 min at 90°C, followed by snap cooling on ice and equilibration at room temperature. Two hundred microliters (10 μg) of murine myelin was pelleted and washed twice in 500 μL binding buffer (145 mM NaCl, 4 mM KCl, 1.5 mM CaCl_2_, 10 mM Na_3_PO_4_).

The pelleted material was then suspended in binding buffer and combined with aptamer (1 μM final concentration) in 100 μL total volume. This mixture was incubated at 37°C for 30 min with rotary mixing. Myelin was again pelleted by centrifugation at 4,700 *g* and washed twice in 1 mL binding buffer to remove any unbound sequences. Remaining bound aptamers were then recovered by solubilization of myelin in 400 μL 2× proteinase K buffer [200 mM Tris-HCl, pH 7.6, 2.5 mM ethylenediaminetetraaceticacid (EDTA), 300 mM NaCl, and 2% sodium dodecyl sulfate] and subsequent phenol/chloroform extraction. This method was repeated over five rounds. Beginning with the third round, nonspecific competitor DNA (100× sheared and denatured salmon testes DNA by mass) was introduced to increase stringency of selection conditions. After five rounds of selection, recovered aptamers were PCR amplified, ligated into the pGEM-Teasy cloning vector, cloned into DH5α cells, colonies grown, plasmids isolated, and aptamer sequences determined by Sanger sequencing.

### Myelin-binding assay

Aptamer binding to myelin suspensions *in vitro* was performed as previously described [22] with freshly prepared murine myelin [18]. Aptamers used in the assay were synthesized (Integrated DNA Technologies) with a 3′ biotin and 5′ 6-FAM label. Labeled aptamers were conjugated to streptavidin by incubation of a 4:1 molar mixture of aptamer:streptavidin with 0.25 μM aptamer at 37°C for 1 h with mixing in PBS supplemented with 1 mM MgCl_2_. Aptamer LJM-3060 is a known G-quadruplex forming molecule used as a negative control in this assay (A_3_GA_2_CA_5_G_2_ATA_3_G_5_AGACG_6_A_2_CATG_4_).

A final concentration of 230 nM aptamer was combined with 0.2 μg/μL murine myelin. Sheared salmon sperm DNA was used in 20-fold excess by mass as a competitor for nonspecific DNA binding to myelin. A final sample volume of 100 μL was incubated at 37°C for 90 min, followed by a 1 min microcentrifugation step to pellet myelin and bound DNA. Supernatant was allocated to a new tube and the pellet was then washed twice with 100 μL PBS. Aliquots of recovered supernatant were combined. The pellet was then resuspended once more in 300 μL PBS so that the volume of myelin-DNA suspensions and recovered supernatants were equal. Fluorescence in these paired samples was read in a black 96-well microplate (Greiner Bio-One) using a plate reader (Analyst AD 96-384) to determine 6-FAM signal and calculate fraction of signal bound to myelin in each sample.

The concentration of aptamer used in this assay was determined based on previously published data [22] and a myelin-binding curve comparing binding of parent molecule LJM-3064 and negative control LJM-5733 (Supplementary Fig. S1). Based on these data, the aptamer concentration used is appropriate for comparison as positive control binding is significantly greater than binding of the negative control, but is below the point of saturation of myelin binding.

### Circular dichroism spectroscopy

Circular dichroism (CD) spectroscopy for analysis of G-quadruplex structures was performed using a JASCO J-810 Spectropolarimeter. Solutions of 4 μM aptamer in 160 mM KCl were heated for 5 min at 90°C, followed by snap cooling on ice and an equilibration period at room temperature before spectra were recorded. Blank spectra for the buffer solution and unconjugated streptavidin in buffer were collected as background measurements that were subtracted from relevant sample readings.

### Gel mobility analysis of G-quadruplex structures

Aptamers were diluted to a final concentration of 4 μM in a buffer containing 10 mM phosphate and 12.5 mM of LiCl, NaCl, KCl, or RbCl. Aptamer solutions were heated at 90°C for 5 min, followed by snap cooling on ice and equilibration at room temperature. Samples were supplemented with loading buffer (Thermo Scientific; R0611) and loaded onto 12% 29:1 acrylamide:bisacylamide gels with 0.5× Tris-borate EDTA running buffer containing 12.5 mM of the same salt solution used for sample preparation. Electrophoresis was for 2.5 h at 300 V (13 V/cm) at room temperature. Images were collected on a Typhoon fluorescence imaging system after SYBR Green I poststain.

### HOG cell-binding assay in culture

Aptamers (1 μM) were conjugated with fluorescein isothiocyanate (FITC)-labeled streptavidin in a 4:1 ratio as described above. HOG cells were grown at 37°C in Dulbecco's modified Eagle medium (DMEM) with supplemented 10% fetal bovine serum (FBS) and Pen/Strep antibiotics (P/S). Culture conditions were controlled at 5% CO_2_, 21% O_2_, and 90% humidity. Aptamer conjugates were added to cells at a final concentration of 1 μM aptamer each in 1 mL fresh DMEM with FBS and P/S. Exposure was for 2 h under standard culture conditions, followed by fixation for 20 min using 10-fold diluted formaldehyde solution (Sigma; 252549). Fixed cells were 4′,6-diamidino-2-phenylindole (DAPI) stained (Roche; 10236276001) for 7 min and washed twice with PBS. Images were collected on a Zeiss LSM 780 microscope using an autofocus routine to capture an optimal image plane based on DAPI channel intensity.

Fluorescence quantitation was performed using an automated image analysis process in CellProfiler. The area of a cell is defined by first selecting nuclei on a DAPI channel image, then expanding all selections by an identical number of pixels in all directions to approximate the space filled by a single cell. FITC channel intensity is then summed over the entire space of each identified cell. This measurement is compared between treatment conditions.

### HOG cell-binding assay by flow cytometry

HOG cells were plated into six-well plates at 30% confluence. Fluorescently labeled oligonucleotide conjugates were prepared by mixing 3′-biotinylated oligonucleotides with FITC-labeled streptavidin (Invitrogen; SA1001) in 4:1 stoichiometric ratio in PBS containing 1 mM MgCl_2_ and incubating at 37°C for 30 min, and storing at 4°C until use. The next day, the medium was aspirated from HOG cells in plates and replaced with 1 mL of fresh medium supplemented with FITC-labeled oligonucleotide-streptavidin complexes at 250 nM final streptavidin concentration. Plates were replaced in the 37°C incubator for 2 h before aspirating the medium, scraping cells into 1 mL fresh medium, and collection of cells by centrifugation at 500 *g* for 5 min at 4°C. Cells were resuspended in 500 μL PBS, placed on ice, and then analyzed by flow cytometry to quantify cell-associated FITC fluorescence.

## Results

### Myelin binding of twenty-nucleotide LJM-3064 derivatives

**FIG. 1.**
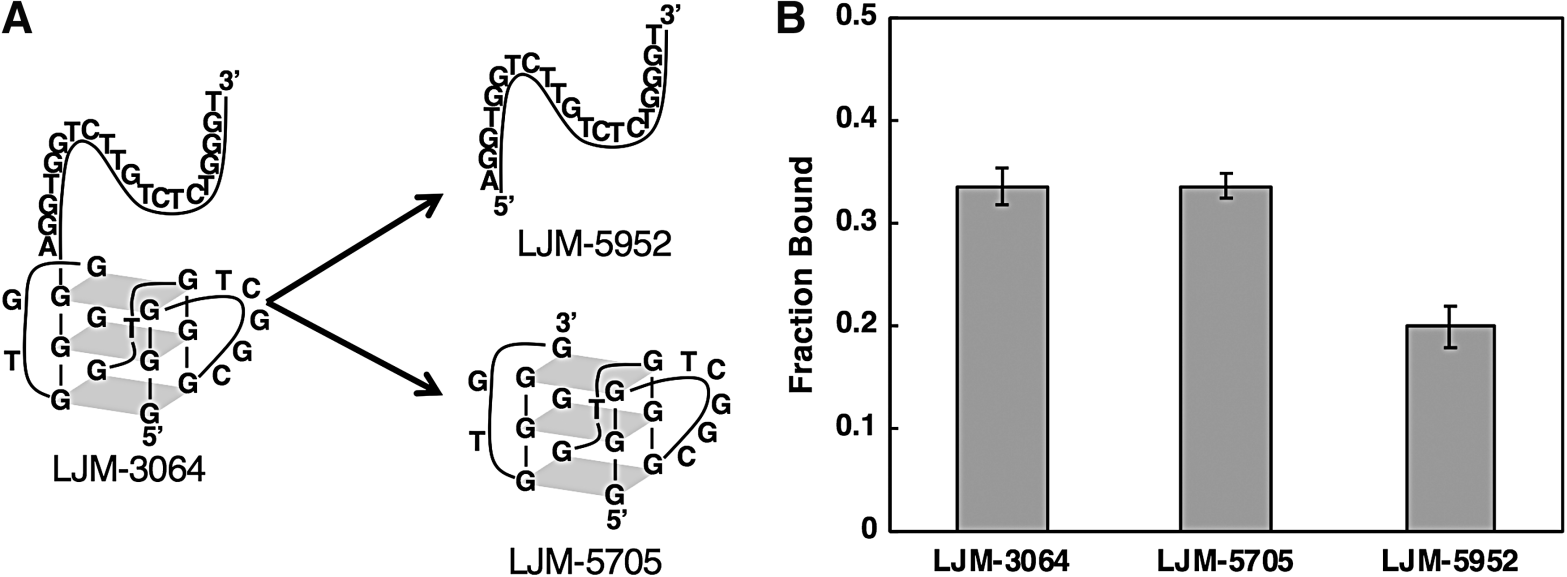
**(A)** Predicted structures of 40-nucleotide antimyelin DNA aptamer LJM-3064 and 20-nucleotide derivatives LJM-5952 and LJM-5705. **(B)** *In vitro* myelin binding by streptavidin conjugates with 3′ biotinylated LJM-3064, LJM-5705, and LJM-5952.

LJM-3064 was previously identified as a strong myelin-binding 40-nucleotide DNA aptamer with a distinct 5′ G-quadruplex-forming half and an unstructured 3′ half [19]. We tested the hypothesis that the myelin-binding activity of LJM-3064 is conferred by the 5′ G-quadruplex-forming half (Fig. 1A). Biotinylated 20-nucleotide derivatives were synthesized as individual DNA oligonucleotides LJM-5705 (5′ G-quadruplex-forming half) and LJM-5952 (3′ unstructured half) and formulated as streptavidin conjugates using a 4:1 aptamer:streptavidin ratio. Using a previously described *in vitro* myelin-binding assay shown to predict *in vivo* remyelinating activity in mice [22], LJM-5705 exhibited myelin binding equivalent to parent aptamer LJM-3064. In contrast LJM-5952 exhibited less myelin binding (Fig. 1B).

Although LJM-5952 binds myelin weakly, this 20 nucleotide sequence does not increase binding of LJM-5705 relative to LJM-3064 and was therefore not included in our optimization. This result confirms that the 20 5′ G-quadruplex forming nucleotides of LJM-3064 are active in myelin binding.

### Optimization of aptamer LJM-5705 results in improved myelin binding

**FIG. 2.**
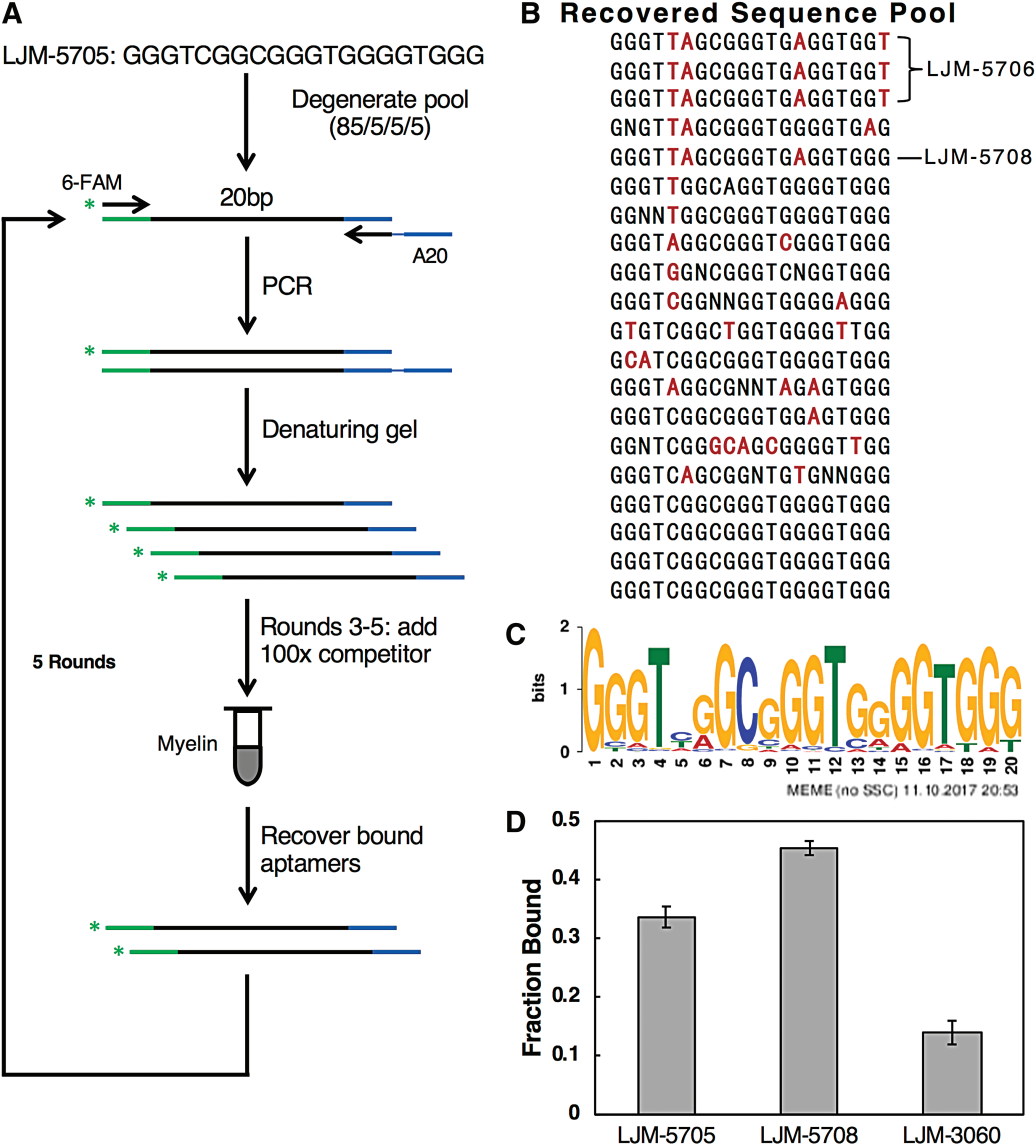
**(A)** Optimization SELEX protocol applied to LJM-5705. **(B)** Recovered sequences following five rounds of optimizing SELEX, where base differences are noted in *red*. **(C)** MEME motif analysis of recovered sequences. **(D)** Myelin-binding properties of streptavidin conjugates of 3′-biotinylated aptamers LJM-5705, LJM-5708, and LJM-3060. SELEX, systematic evolution of ligands by exponential enrichment. Color images are available online.

We next assessed whether sequence modifications within 20-nucleotide guanosine-rich aptamer LJM-5705 could yield enhanced myelin-binding properties. A SELEX strategy similar to that previously described [18] was used to optimize the sequence of LJM-5705. Our approach involved synthesis of a lightly randomized degenerate SELEX library derived from the LJM-5705 sequence, with 15% probability of base randomization at each nucleotide position (Fig. 2A). Through iterative selection of this library for binding to suspensions of crude murine myelin *in vitro* and recovery of bound sequences in each cycle, the best myelin-binding molecules emerged. These optimized DNAs included sequences that outperformed the parental aptamer for myelin-binding capabilities. In rounds 3–5 of SELEX, a 100-fold mass excess of sheared and denatured salmon sperm DNA was added as a competitor for nonspecific DNA-binding activity. After five rounds, the recovered pool was sequenced, and candidate optimized antimyelin aptamers were identified (Fig. 2B).

MEME motif analysis [23] of the recovered pool indicated that bases involved in predicted G-quadruplex formation were highly conserved among the recovered molecules, but the loop regions separating guanosine-rich sequences displayed higher mutation frequency (Fig. 2C). It is notable that 12/20 sequences showed a switch from C to another base at the fifth position, with transition to thymidine being the most common (7/12). Similarly, 6/20 sequences contained a change at the sixth position, exclusively mutating to adenine. In addition, 5/20 bases differed at the 14th position, with a strong preference for adenine (4/5). Each of these changes is predicted to alter loops separating G-tetrad stacks, likely impacting binding properties without perturbing of the core G-quadruplex. One of the optimized molecules, LJM-5708, was selected for further *in vitro* testing to assess myelin-binding properties. Analysis was carried out with a standard myelin-binding assay [22]. Comparing 20-nucleotide LJM-5708 to the parental 20-nucleotide aptamer LJM-5705, we observe a statistically significant enhancement in myelin binding for LJM-5708. This suggests that the SELEX approach was successful in identifying a variant with improved binding to the crude myelin suspension.

### G-quadruplex structure is essential for myelin-binding activity

**FIG. 3.**
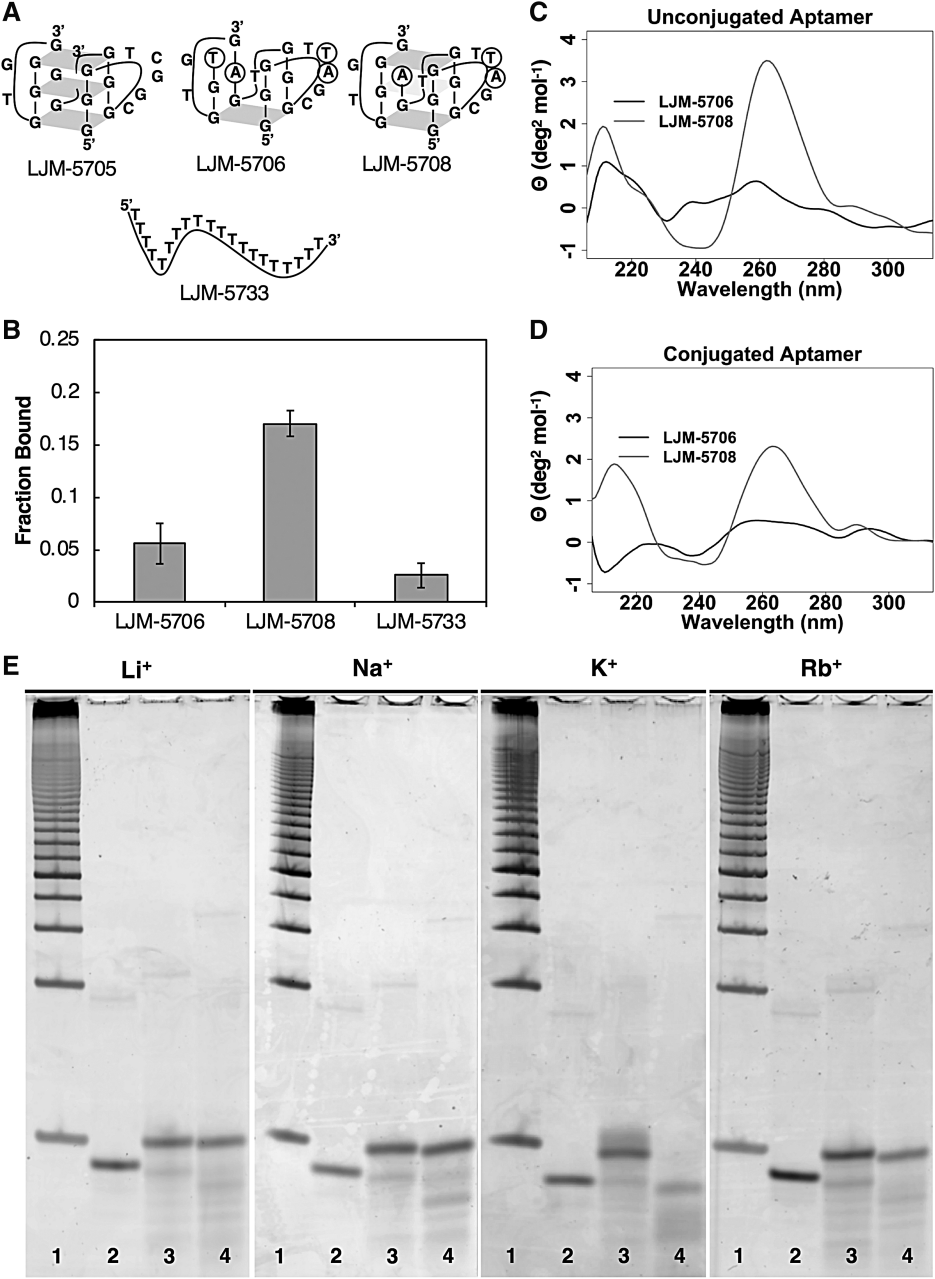
**(A)** Predicted structures of aptamers of interest. *Circled* bases in LJM-5706 and LJM-5708 indicate differences from LJM-5705. **(B)** Fraction of each biotinylated aptamer bound as a streptavidin conjugate in myelin-binding assay. **(C)** CD spectra of folded LJM-5706 and LJM-5708 in solution. **(D)** CD spectra of folded biotinylated LJM-5706 and LJM-5708 conjugated to streptavidin (CD spectra of streptavidin subtracted). **(E)** Polyacrylamide electrophoretic gel mobility assay of aptamers under the indicated ionic conditions. Lanes are: 1, duplex DNA reference ladder; 2, LJM-5733; 3, LJM-5706; 4, LJM-5708. CD, circular dichroism.

Among the variants identified by optimization SELEX was LJM-5706, identical to LJM-5708 except for mutation of the 3′ terminal guanine nucleotide to thymine (Fig. 3A). Because this position is predicted to participate in a G-tetrad, this result raised the possibility that an incomplete G-quadruplex structure might be tolerated for myelin binding. However, LJM-5706 displayed very low myelin-binding capacity compared to LJM-5708 (Fig. 3B). This suggests that loss of G-quadruplex formation disables myelin binding.

We further characterized G-quadruplex formation by LJM-5708 (active) and LJM-5706 (inactive). CD spectroscopy confirmed that LJM-5708 forms a parallel-stranded G-quadruplex in phosphate buffer supplemented with 160 mM KCl, indicated by a positive peak in molar ellipticity at 260 nm and a negative peak at 240 nm (Fig. 3C and Supplementary Table S1) [24].

These findings are consistent with the previously reported spectra of LJM-3064, the 40 nucleotide parent molecule, which forms the same G-quadruplex [19]. LJM-5706, however, did not form a similar structure under these conditions. Since the *in vitro* myelin-binding assay and *in vivo* remyelination studies typically utilize streptavidin-conjugated biotinylated aptamer formulations, we further characterized the folded structures of 3′-biotinylated LJM-5706 and LJM-5708 when conjugated to streptavidin (Fig. 3D). The parallel-stranded G-quadruplex structure of LJM-5708 remains stable when the aptamer is tethered to streptavidin. LJM-5708 also forms a stable G-quadruplex in selection buffer, but LJM-5706 has a significantly less stable secondary structure (Supplementary Fig. S2; Supplementary Table S1).

To further confirm the G-quadruplex structure formed by LJM-5708, we performed electrophoretic gel mobility analysis in the presence of various monovalent cations, including Li^+^, Na^+^, K^+^, and Rb^+^. G-quadruplex structures are preferentially stabilized by K^+^ ions, resulting in a more compact structure with increased electrophoretic mobility [25–27]. The results are shown in Fig. 3E. LJM-5708 (Fig. 3E, lane 4) is indeed preferentially stabilized by K^+^ compared to other monovalent cations, consistent with the formation of G-quadruplex structure. In contrast, the mobility of LJM-5706 (Fig. 3E, lane 3) did not change relative to unstructured control dT_20_ (Fig. 3E, lane 2), or duplex DNA ladder (lane 1). The relative G-quadruplex stability of each aptamer in various ionic conditions was further confirmed by CD spectroscopy (Supplementary Fig. S2; Supplementary Table S1).

### Preservation of myelin-binding specificity in different protein conjugates

To date we have applied conjugation of biotinylated aptamers to streptavidin to form multimers for remyelinating therapy in living mice [18]. Experiments showed that streptavidin conjugation formation was essential for enhanced biodistribution and aptamer-mediated remyelination in mice [20]. Unconjugated aptamer was not effective. This necessity for protein conjugation is not understood, although multimerization may enhance target avidity, as has been described previously [28–30]. It is also possible that aptamer conjugation to protein creates a more favorable overall charge density affecting biodistribution and target affinity. Other advantages unrelated to target binding may be conferred by conjugation to streptavidin, such as resistance to nuclease activity by terminal modifications. Larger streptavidin-aptamer complexes could be more resistant to renal clearance than free aptamers, as addressed in some instances by terminal polyethylene glycol modifications to enhance duration of circulation [31]. These concerns and others, such as immunogenicity of aptamer complexes, are well-documented challenges for *in vivo* applications of aptamers [32].

**FIG. 4.**
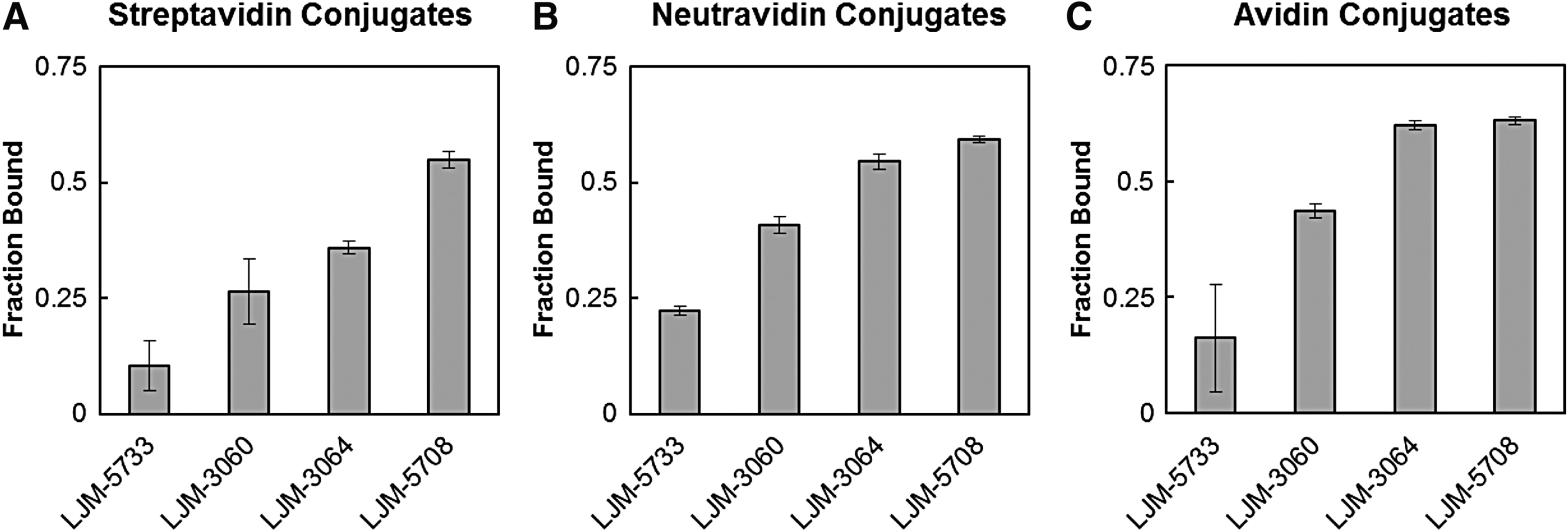
Fraction of biotinylated aptamer bound to myelin after conjugation to **(A)** streptavidin, **(B)** neutravidin, or **(C)** avidin.

Given the importance of protein conjugation, we compared myelin binding for three common biotin-binding tetramers: streptavidin, neutravidin, and avidin. Streptavidin is a bacterial biotin-binding tetramer with a moderate isoelectric point and low nonspecific binding. Neutravidin is a deglycosylated form of egg white avidin. Streptavidin-aptamer complexes bound myelin when conjugated with LJM-5708, while negative controls LJM-3060 and LJM-5733 bound poorly (Fig. 4A). When neutravidin from egg white was substituted for bacterial streptavidin, myelin binding by negative control molecules was significantly increased. The difference in binding activity between conjugates of LJM-3064 and LJM-5708 remained statistically significant (*p* < 0.05; Fig. 4B). Finally, in complexes with avidin (Fig. 4C) binding differences between LJM-3064 and the optimized aptamer LJM-5708 were no longer significant. These results suggest that the identity of the biotin-binding protein used as a conjugation core influences the specificity of the myelin-binding interaction. Effects of the identity of the conjugation protein on remyelination *in vivo* will be the subject of future studies.

### Myelin-specific aptamers bind HOG cells

**FIG. 5.**
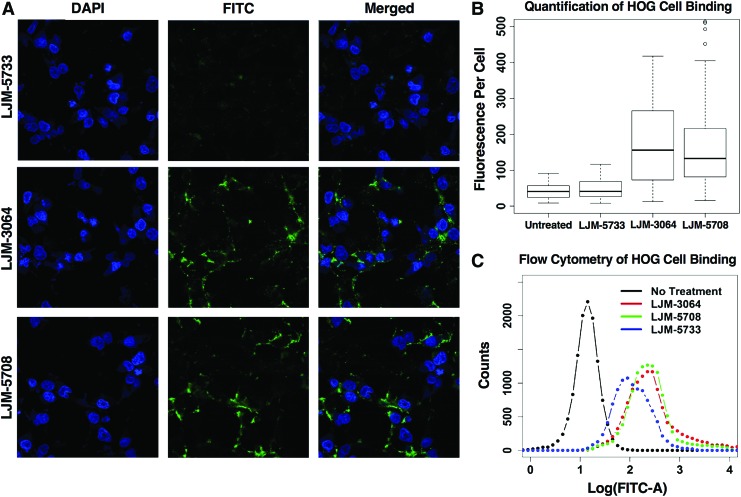
**(A)** Confocal microscopy images of aptamer-streptavidin conjugate binding to HOG cells. **(B)** Quantification of binding of aptamer-streptavidin complexes. **(C)** Histogram of flow cytometry analysis of binding of aptamer-streptavidin complexes. HOG, human oligodendroglioma. Color images are available online.

Finally, we assessed the degree to which streptavidin conjugates of antimyelin DNA aptamers bind the surface of HOG cells under typical cell culture conditions. The results are shown in Fig. 5. Streptavidin complexes of both LJM-3064 and LJM-5708 are capable of strong HOG cell binding relative to controls. Binding was assessed by both confocal microscopy with image quantification (Fig. 5A, B) and flow cytometry (Fig. 5C) with similar results.

## Discussion

In this study, we use both rational and combinatorial approaches to optimize a DNA aptamer with potential therapeutic application in MS. The ability to exploit combinatorial selections differs from the conventional process of lead compound identification and optimization in a typical small molecule drug development pipeline. The latter involves candidate compound identification, the creation of a limited library of structural permutations, and screening. Our SELEX-based approach to aptamer optimization allows for simultaneous competition of ∼10^14^ degenerate molecules in a single test tube, giving ∼10^9^-fold improvement in diversity beyond what is achievable in a small molecule library with the efficiency of handling a single sample. This powerful approach to optimization of a lead demonstrates an intrinsic advantage of nucleic acid drug discovery and optimization efforts.

Analysis of recovered sequence pools from our aptamer optimization of the 20-nucleotide aptamer LJM-5705 yielded the enhanced aptamer LJM-5708. The 5′ half of the original 40-nucleotide aptamer (LJM-3064) showed strong convergent evolution identifying three nucleotides that differ from LJM-3064. Such strong convergence indicates that the optimized 20-nucleotide molecule (LJM-5708) is an improved myelin-binding aptamer within the constraints of the SELEX design (ie, degenerate library based on LJM-5705, 20 nucleotide length restriction, and variation limited to base substitutions of the 4 natural DNA nucleotides without insertions or deletions).

Indeed, we demonstrate that optimized antimyelin aptamer LJM-5708 has enhanced myelin-binding properties relative to the parental aptamer. LJM-5708 retains the G-quadruplex secondary structure of the parent molecule, which appears to be essential for strong myelin-binding activity. G-quadruplex-forming aptamers are not uncommon results of SELEX experiments using DNA libraries [33–35], and many well-documented examples of G-quadruplex–protein interactions exist in nature [36–38]. MEME motif analysis of available SELEX data from the current work demonstrates that bases implicated in G-quadruplex formation are highly conserved among recovered molecules following optimization SELEX. Our SELEX data do not allow us to determine the balance of target recognition between the overall shape of the G-quadruplex, and the role of specific functional groups on displayed DNA elements. It is reasonable to assume that the G-quadruplex scaffold plays an essential role in displaying a three-dimensional array of specific functional groups for high affinity to the, as yet unidentified, molecular target on myelin.

Importantly, we also confirm that antimyelin aptamers LJM-3064 and LJM-5708 both have the ability to bind to cultured HOG cells. This is important because it is the first direct evidence of aptamer binding to an intact myelin-rich cell surface. The result also demonstrates that antimyelin aptamers selected against mouse myelin recognize HOG cells. These confocal microscopy and flow cytometry results are the first confirmation of cross-species molecular recognition of myelin by remyelinating DNA aptamers.

Mouse-derived myelin suspensions, used in the myelin-binding assay, and human myelin sheaths, as studied in HOG cells, are of relatively similar composition. The classical human myelin proteins 2′ 3′-cyclic-nucleotide 3′-phosphodiesterase (CNP), myelin-associated glycoprotein (MAG), myelin basic protein (MBP), myelin oligodendrocyte glycoprotein (MOG), oligodendrocyte specific protein (OSP), and myelin proteolipid protein (PLP) make up more than 90% of the rodent myelin proteome, although the remaining fraction does contain a range of proteins unique to rodent myelin [39]. A total of 678 myelin-associated proteins have been identified in human myelin, compared to 475 in rodents, 308 of which are shared by the 2. The bulk characteristics of human and mouse myelin are comparable for study of MS and other myelin-associated diseases [39]. Nonsimilar proteins make up a relatively small fraction of each organism's myelin sheath.

Our study of protein conjugates indicates that bacterial streptavidin complexes bind myelin with the greatest specificity. Negative control aptamer complexes assembled with egg white avidin or neutravidin display higher background binding than with streptavidin. Although streptavidin, neutravidin, and avidin all bind biotin with extreme affinity, their structural differences likely are responsible for varied myelin specificity of conjugated DNA aptamers.

About 10% of the total mass of avidin is contributed by charged carbohydrate modifications, yielding an isoelectric point (pI) of 10–10.5 [40]. This means that avidin is strongly positively charged at neutral pH. Myelin, primarily a lipid suspension (pI ∼2), is strongly negatively charged at physiological pH [41]. Thus, electrostatic interactions may explain increased nonspecific myelin binding by avidin-aptamer complexes. Neutravidin (deglycosylated avidin) is less cationic than avidin [40], but more charged than bacterial streptavidin. Besides concerns about the potential immunogenicity of bacterial streptavidin, this protein harbors an RYD peptide sequence reported to mimic the RGD fibronectin recognition sequence [42]. Streptavidin conjugates may therefore interact with the extracellular matrix *in vivo*. Such interactions may be relevant to the known remyelination activity of antimyelin aptamer streptavidin conjugates [18].

## Conclusions

Our results indicate that the 5′ 20 nucleotides of previously identified DNA 40-nucleotide remyelinating aptamer LJM-3064 preserve a parallel G-quadruplex structure associated with myelin binding activity. This isolated 20-nucleotide aptamer shows strong myelin-binding activity that was increased significantly by further *in vitro* selection. The G-quadruplex secondary structure is retained in optimized DNA aptamer LJM-5708, although one of three base changes alters the central G-tetrad. Myelin-binding assays with biotinylated aptamers conjugated to three different core proteins suggest that streptavidin enhances binding specificity. Finally, a HOG cell-binding assay shows that streptavidin conjugates of optimized antimyelin DNA 20-nucleotide aptamer LJM-5708 bind oligodendrocytes comparably to the parent 40-nucleotide sequence. This evidence of cross-species specificity for myelin is a key component in the nomination of LJM-5708 as a lead molecule for further investigation. Optimized 20-nucleotide DNA aptamer LJM-5708 is thus a strong candidate for further preclinical testing, including pharmacokinetic analysis and remyelination assay *in vivo* [18,20].

## Supplementary Material

Supplemental data
