# Supplementary material for: Optimization of a 40-mer Antimyelin DNA Aptamer Identifies a 20-mer with Enhanced Properties for Potential Multiple Sclerosis Therapy
Source: Nucleic Acid Ther. 2019 May 30;29(3):126–35. doi: 10.1089/nat.2018.0776 (PMC6555174; doi:10.1089/nat.2018.0776)

**Supplemental data**

# Optimization of a 40-mer anti-myelin DNA aptamer identifies a 20-mer with enhanced properties for potential multiple sclerosis therapy

Brandon Wilbanks[1*], John Smestad[1,2*], Robin M. Heider[1], Arthur E. Warrington[3], Moses Rodriguez[3], and L. James Maher, III[1]

1. Department of Biochemistry and Molecular Biology, Mayo Clinic College of Medicine and Science, 200 First Street SW, Rochester, MN 55905, USA

2. Medical Scientist Training Program, Mayo Clinic College of Medicine and Science, 200 First Street SW, Rochester, MN 55905, USA

3. Departments of Neurology and Immunology, Mayo Clinic College of Medicine and Science, 200 First Street SW, Rochester, MN 55905, USA

*Authors contributed equally to this work.

**Supplemental Table 1**

**Summary of molar ellipticity values from CD spectroscopy for aptamers LJM-5706 and LJM-5708 in various ionic conditions.**

| | Θ ($deg^2\ mol^{-1}$), 240 nM | Θ ($deg^2\ mol^{-1}$), 260 nM |
|---|---|---|
| LJM-5706, 160 Mm $Li^+$ | 0.0515 | -0.199 |
| LJM-5706, 160 Mm $Na^+$ | -0.723 | 0.114 |
| LJM-5706, 160 Mm $K^+$ | 0.0976 | 0.851 |
| LJM-5706, 160 Mm $Rb^+$ | -0.935 | 1.338 |
| LJM-5706, Selection Buffer | -0.981 | 1.541 |
| LJM-5706:Streptavidin, 160 Mm $K^+$ | -0.369 | 0.481 |
| LJM-5708, 160 Mm $Li^+$ | 0.051 | -0.199 |
| LJM-5708, 160 Mm $Na^+$ | -0.317 | 0.366 |
| LJM-5708, 160 Mm $K^+$ | -0.953 | 3.778 |
| LJM-5708, 160 Mm $Rb^+$ | -0.0926 | 0.324 |
| LJM-5708, Selection Buffer | -0.732 | 3.899 |
| LJM-5708:Streptavidin, 160 Mm $K^+$ | -0.478 | 2.208 |

**Supplemental Figure 1: Myelin binding of aptamers LJM-3064 and LJM-5733 under standard myelin binding assay conditions.**

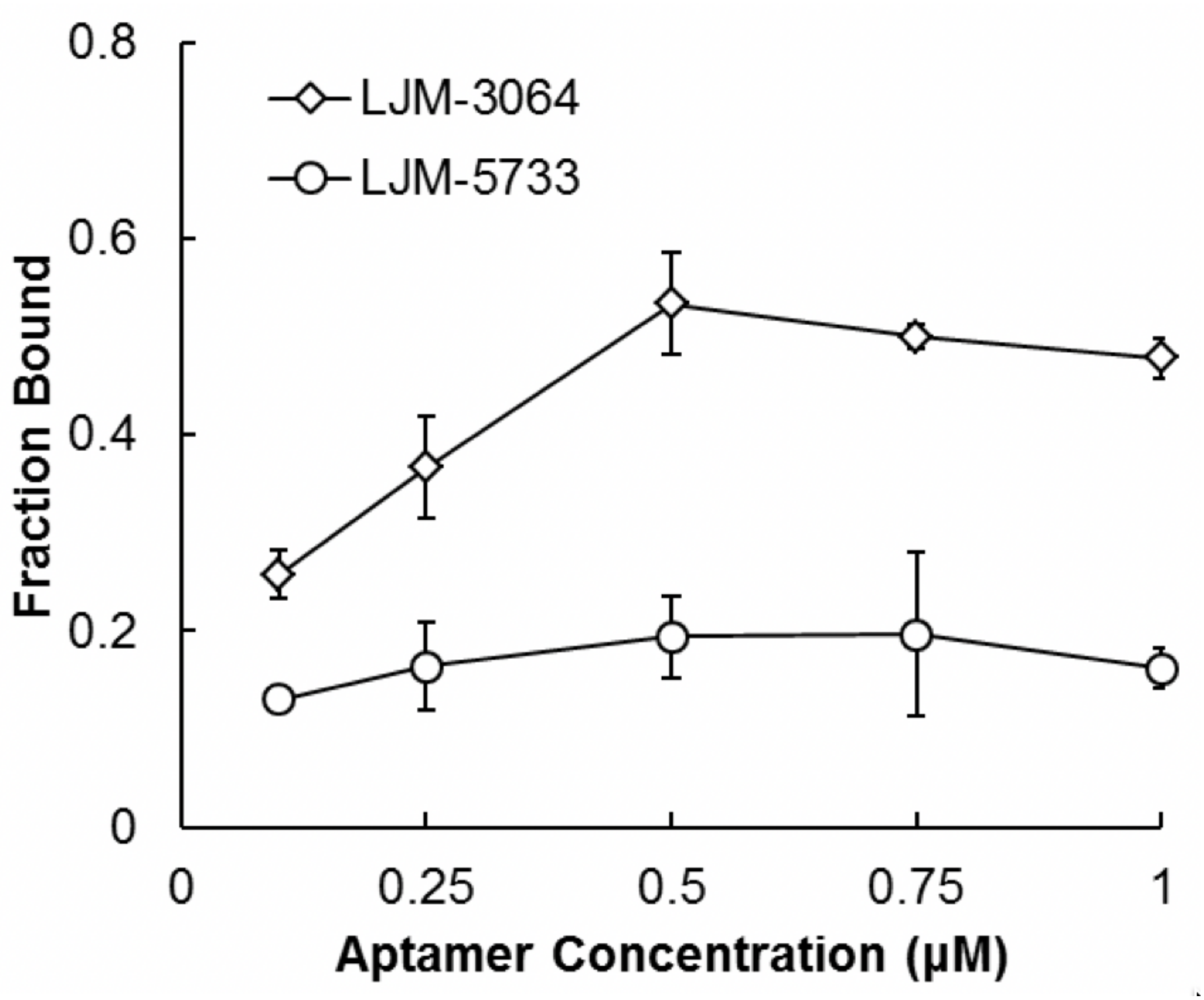

**Supplemental Figure 2: CD spectroscopy analysis of folded A) LJM-5706 and B) LJM-5708 in 160 mM $Li^+$, $Na^+$, $Rb^+$, or selection buffer reveals that LJM-5708 forms a stable G-quadruplex in optimization SELEX conditions.**

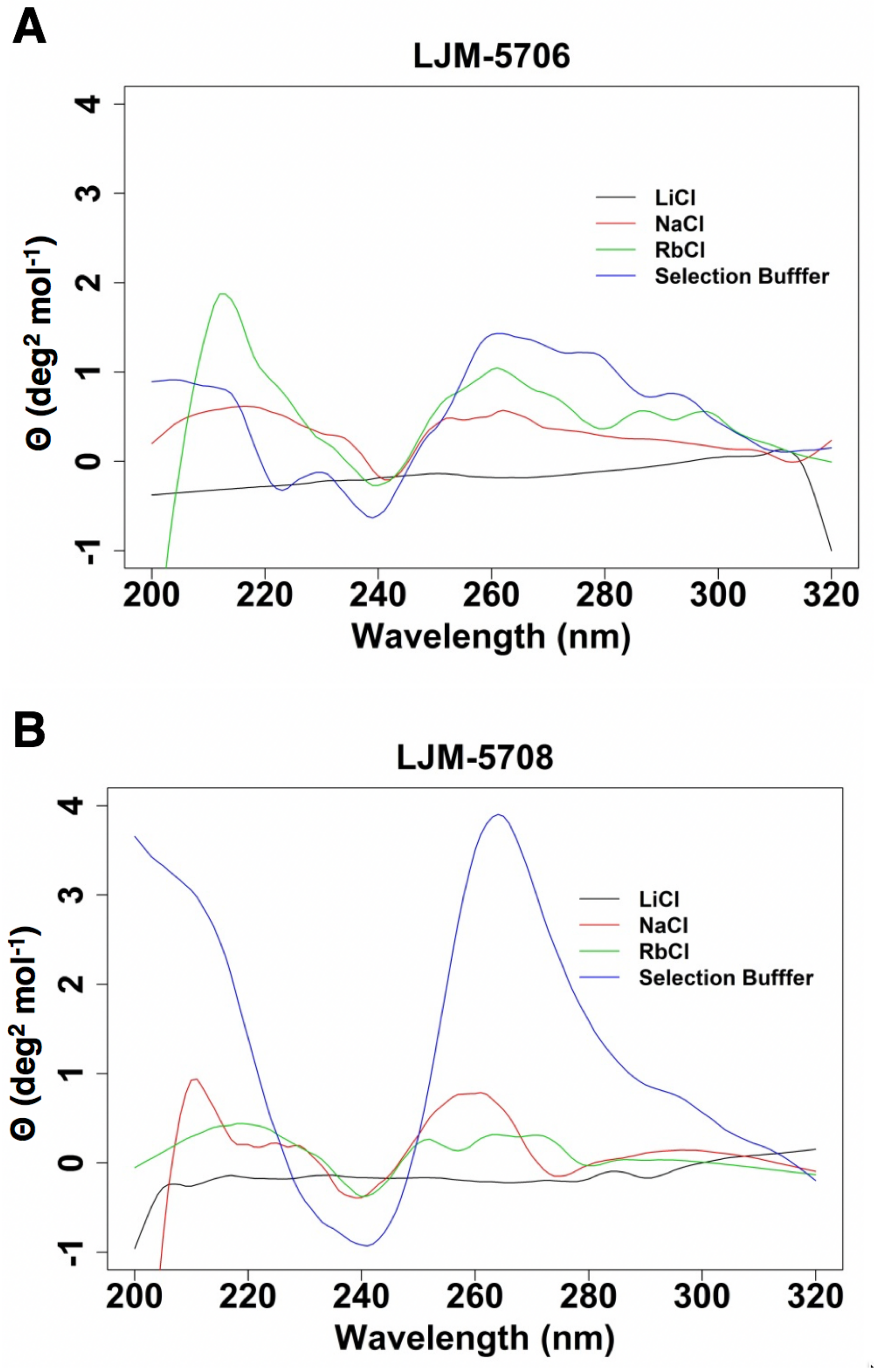

Supplement: Supplemental data [file Supp_Data.pdf]
